# Differential Antivenom and Small-Molecule Inhibition of Novel Coagulotoxic Variations in *Atropoides, Cerrophidion, Metlapilcoatlus,* and *Porthidium* American Viperid Snake Venoms

**DOI:** 10.3390/toxins14080511

**Published:** 2022-07-26

**Authors:** Lee Jones, Nicholas J. Youngman, Edgar Neri-Castro, Alid Guadarrama-Martínez, Matthew R. Lewin, Rebecca Carter, Nathaniel Frank, Bryan G. Fry

**Affiliations:** 1 Venom Evolution Lab, School of Biological Sciences, University of Queensland, St Lucia, QLD 4072, Australia; n.youngman@uq.edu.au; 2 Departamento de Medicina Molecular y Bioprocesos, Instituto de Biotecnologia, Universidad Nacional Autónoma de México, Av. Universidad 2001, Cuernavaca 62210, Mexico; nericastroedgare@gmail.com (E.N.-C.); alid.guadarrama@ibt.unam.mx (A.G.-M.); 3 Ophirex Inc., Corte Madera, CA 94925, USA; matt@ophirex.com (M.R.L.); rebecca@ophirex.com (R.C.); 4 MToxins, 717 Oregon Street, Oshkosh, WI 54902, USA; nate@mtoxins.com

**Keywords:** antivenom, procoagulant, pseudo-procoagulant, anticoagulant

## Abstract

Within Neotropical pit-vipers, the Mexican/Central-American clade consisting of *Atropoides*, *Cerrophidion*, *Metlapilcoatlus*, and *Porthidium* is a wide-ranging, morphologically and ecologically diverse group of snakes. Despite their prevalence, little is known of the functional aspects of their venoms. This study aimed to fill the knowledge gap regarding coagulotoxic effects and to examine the potential of different therapeutic approaches. As a general trait, the venoms were shown to be anticoagulant but were underpinned by diverse biochemical actions. Pseudo-procoagulant activity (i.e., thrombin-like), characterized by the direct cleavage of fibrinogen to form weak fibrin clots, was evident for *Atropoides picadoi, Cerrophidion* *tzotzilorum*, *Metlapilcoatlus mexicanus, M. nummifer, M. occiduus, M. olmec*, and *Porthidium porrasi.* In contrast, other venoms cleaved fibrinogen in a destructive (non-clotting) manner, with *C. godmani* and *C. wilsoni* being the most potent. In addition to actions on fibrinogen, clotting enzymes were also inhibited. FXa was only weakly inhibited by most species, but *Cerrophidion godmani* and *C. wilsoni* were extremely strong in their inhibitory action. Other clotting enzymes were more widely inhibited by diverse species spanning the full taxonomical range, but in each case, there were species that had these traits notably amplified relatively to the others. *C. godmani* and *C. wilsoni* were the most potent amongst those that inhibited the formation of the prothrombinase complex and were also amongst the most potent inhibitors of Factor XIa. While most species displayed only low levels of thrombin inhibition, *Porthidium dunni* potently inhibited this clotting factor. The regional polyvalent antivenom produced by Instituto Picado Clodomiro was tested and was shown to be effective against the diverse anticoagulant pathophysiological effects. In contrast to the anticoagulant activities of the other species, *Porthidium volcanicum* was uniquely procoagulant through the activation of Factor VII and Factor XII. This viperid species is the first snake outside of the *Oxyuranus/Pseudonaja* elapid snake clade to be shown to activate FVII and the first snake venom of any kind to activate FXII. Interestingly, while small-molecule metalloprotease inhibitors prinomastat and marimastat demonstrated the ability to prevent the procoagulant toxicity of *P. volcanicum*, neither ICP antivenom nor inhibitor DMPS showed this effect. The extreme variation among the snakes here studied underscores how venom is a dynamic trait and how this can shape clinical outcomes and influence evolving treatment strategies.

## 1. Introduction

Snakebite envenomation remains a globally significant issue that continues to be neglected. In Central America, an estimated 5500 venomous snakebites occur each year [1], while 4000 venomous bites are reported in Mexico [2]. However, this is likely a vast underestimation due to data deficiencies, with many rural populations typically not receiving treatment or recording snakebites [3]. The lack of treatment leads to severe local effects and potentially permanent sequalae as a result of envenomation [4,5]. Regardless of data gaps, the existing statistics indicate that in the Americas, pit-vipers are responsible for the vast majority of snakebites [3,5].

Within Neotropical pit-vipers, a clade consists of the morphologically and ecologically distinct genera *Atropoides, Cerrophidion, Metlapilcoatlus,* and *Porthidium*. These snakes are found from eastern, southeastern, and southwestern Mexico to western Panama, with only *Porthidium* extending into northern South America [6]. *Atropoides* and *Metlapilcoatlus*, jumping pit-vipers, are heavy-bodied species that occur in tropical and subtropical habitats [6,7]. The montane pit-viper *Cerrophidion* species are moderately stout, high-altitude specialists, being found between 1300 and 3500 m in elevation [8]. *Porthidium* species live in low–middle-elevation forests and are typically much more slender than *Atropoides, Cerrophidion,* and *Metlapilcoatlus* species [9].

Due to the cryptic nature of these species, very little is known about their natural history, including diet and the functional aspects of their venom. Much of what is known of the venom consists of proteomics investigations, which have revealed that venom composition between species is relatively similar and is typically dominated by metalloproteinases (SVMP), kallikrein-type serine proteases (SVSP), and phospholipases A_2_ (PLA2s) [10,11,12,13,14,15,16]. However, as these toxin types are multifunctional, such proteomic venom-composition studies do not shed light upon venom activity. Prior work has demonstrated an overall anticoagulant effect for *Cerrophidion sasai* (formerly considered a Costa Rican population of *Cerrophidion godmani* before being elevated to full species status), *Porthidium nasutum,* and *Porthidium ophryomegas* [13,17]. Nevertheless, these studies have not elucidated the biochemical mechanisms leading to the net anticoagulant state. Similarly, work showing that *Metlapilcoatlus* venom has a clotting action on plasma does not distinguish between true procoagulant activity (characterized by the activation of endogenous clotting factors leading to well-ordered fibrin clot) and pseudo-procoagulant/”thrombin-like” activity (direct action on fibrin leading to weak, transient fibrin clots) [16,18]. Consequently, the lack of research regarding the functional effects of venom not only limits the evolutionary understanding of the selection pressures acting on these species but also presents a significant knowledge gap in the effective clinical management of snakebite.

As a result of their wide distribution and proximity with humans in forests and agricultural lands, these species are likely to cause a considerable number of bites in the regions in which they occur. For example, *Porthidium lansbergii* alone is thought to contribute to 20% of snakebites in Colombia [11]. Indeed, Gutiérrez [1] has long emphasized a critical need to characterize the functional aspects of *Atropoides, Cerrophidion, Metlapilcoatlus,* and *Porthidium* venoms in order to understand species-specific clinical manifestations of snakebite-envenoming syndromes, which may vary drastically despite the proteomic similarity of the venoms. In addition, bites from coagulotoxic snakes within these regions are typically diagnosed as “bothropic envenomation” [1,19]. This may underestimate the true number of snakebites these species are responsible for due to the misidentification of the offending snakes. Therefore, there is a significant gap regarding the specific venom effects of medically relevant snakes in this clade, including how well the regional antivenoms work against their venoms. In addition, as *Bothrops* species are unique amongst American pit-vipers as having potent procoagulant toxicity (Factor X activation and prothrombin activation [20]), using “bothropic envenomation” as a catch-all term may obscure extreme coagulotoxicity variations in venom action in non-*Bothrops* viperid species while obscuring the impact of non-*Bothrops* species.

Pit-viper-snakebite treatment in Central America typically involves the use of a polyvalent equine antivenom, PoliVal-ICP, made by Instituo Clodomiro Picado in Costa Rica. This antivenom is produced using an immunizing mixture based on equal amounts of venoms of *Bothrops asper* (Caribbean and Pacific populations), *Crotalus simus*, and *Lachesis stenophrys* [21,22]. Testing venoms that are not included in the immunizing mixture reveals the cross-reactivity of the ICP antivenom. Due to the high diversity of species within the region, the degree of cross-reactivity can indicate the efficacy of the antivenom as a successful treatment between species, which is particularly important when the offending snake has not been identified beyond being a viperid species. While limited in scope, prior research into cross-reactivity has yielded positive results, showing strong ability to neutralize venom effects of Central American species *A. picadoi*, *C. sasai*, *M. mexicanus*, *P. nasutum*, and *P. ophryomegas* [17,18,22,23], while reports in Mexico have indicated that the two Mexican antivenoms are not effective in neutralizing the lethality of the juvenile and adult venoms of *M. nummifer* (>24.6 and 33.3 mgAV/mgV) [16].

This study comprised two main aims. The first aim was to characterize the functional effects of *Atropoides*, *Cerrophidion*, *Metlapilcoatlus*, and *Porthidium* venoms upon the human coagulation cascade. It was hypothesized that such a diverse clade of snakes would incur a variety of venom effects. Secondly, we wanted to test the efficacy of the regionally widely used PoliVal-ICP antivenom against medically significant species, as none of the studied species are included in the antivenom immunizing mixture. The results of this study comprise the most complete assessment of the functional activity of *Atropoides*, *Cerrophidion*, *Metlapilcoatlus*, and *Porthidium* venoms to date.

## 2. Results

**Figure 1:**
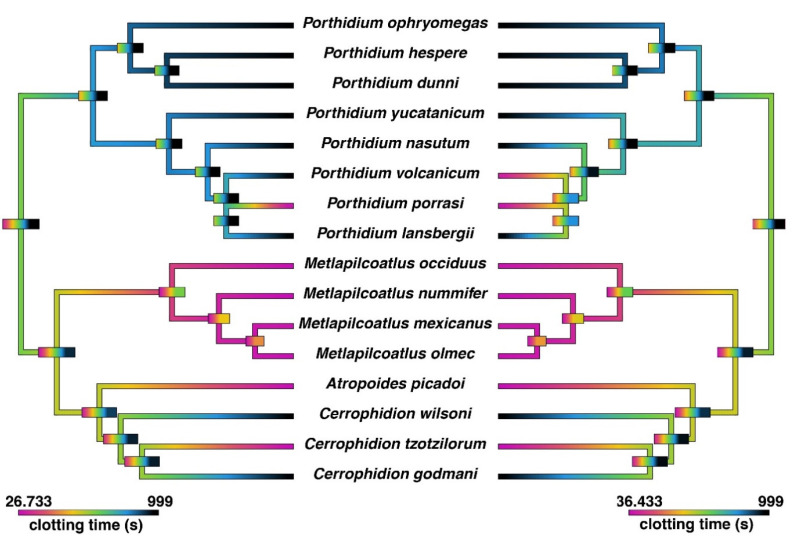
**Ancestral state reconstruction of coagulotoxic venom effects on human fibrinogen (left) and plasma (right).** The spontaneous clotting time of human plasma was 358.9 s. Cooler colors represent the inhibition of clotting in plasma (anticoagulant effect) and no clotting of fibrinogen. Warmer colors represent clotting in plasma and fibrinogen. The maximum machine reading time was 999 s. Bars represent 95% confidence intervals for the estimate at each node. The phylogeny used is based upon Alencar et al. [24] and timetree.org. The correlations between fibrinogen clotting and plasma clotting were congruent with direct action upon fibrinogen rather than the activation of clotting factors. However, for *P. volcanicum*, only plasma was clotted, with no direct action upon fibrinogen, which was suggestive of the activation of one or more clotting factors.

Initial tests on human plasma revealed that accelerated clotting was evident for *Atropoides picadoi*, *Cerrophidion tzotzilorum*, *Metlapilcoatlus mexicanus, M. nummifer*, *M. occiduus*, *M. olmec*, *Porthidium porrasi*, and *P. volcanicum*. As simple clotting tests cannot distinguish between clotting due to factor-activation that produces strong, well-ordered fibrin clots (true procoagulant toxicity) or to the direct “thrombin-like” cleavage of fibrinogen to produce weak, aberrant fibrin clots (pseudo-procoagulant toxicity), additional tests were run for the ability to directly clot fibrinogen. All these species except for *P. volcanicum* were able to directly clot fibrinogen (Figure 1).

**Figure 2:**
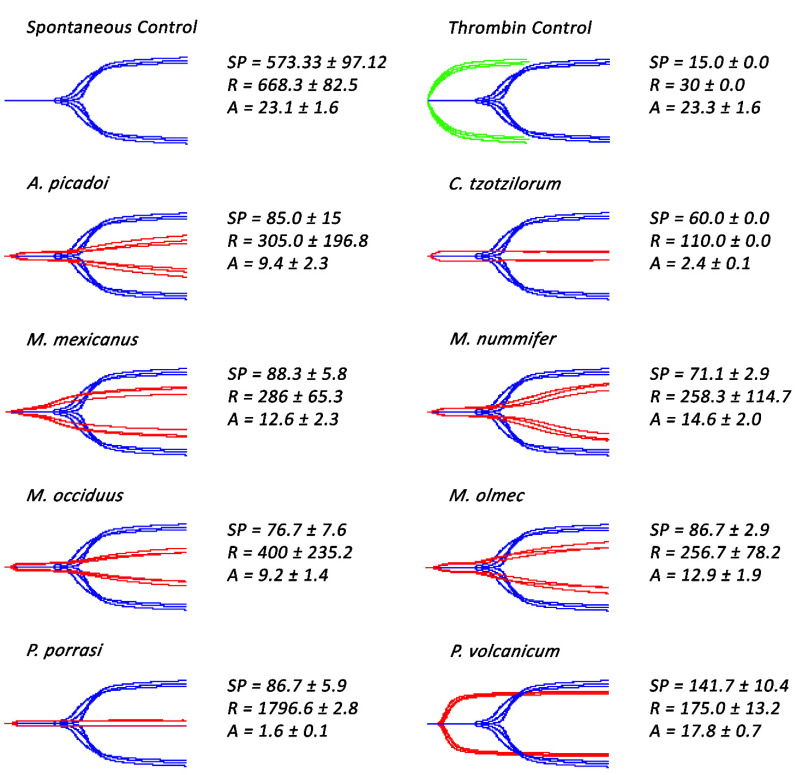
**Thromboelastography traces showing *Atropoides/Cerrophidion/Metlapilcoatlus* venoms ability to clot human plasma in comparison to the spontaneous control.** Samples showed to possess pseudo-procoagulant (*Atropoides*/*Cerrophidion*/*Metlapilcoatlus*) and procoagulant (*P. volcanicum*) venoms. Blue traces represent spontaneous controls, green traces represent thrombin control, and red traces represent samples incubated with venoms. SP = split point, which is the time taken until the clot began to form (min). R = time to initial clot formation, where formation is 2 mm + (min). A (amplitude) = clot strength (mm). Assays were performed in triplicate (*n* = 3) with data representing the mean ± SD.

Subsequent thromboelastographic studies provided a distinction between pseudo-procoagulant (weak clots formed by the direct “thrombin-like” action on fibrinogen) and true procoagulant (strong clots formed by the activation of clotting-factor zymogens upstream of fibrinogen) venom activities. Weak clots were confirmed for *Atropoides picadoi, Cerrophidion tzotzilorum*, *Metlapilcoatlus mexicanus, M. nummifer, M. occiduus, M. olmec*, and *Porthidium porrasi* (Figure 2), revealing that these venoms clotted via the pseudo-procoagulant (‘thrombin-like’) pathway. In contrast, strong clots were observed in *P. volcanicum*, suggestive of a true procoagulant activity.

**Figure 3:**
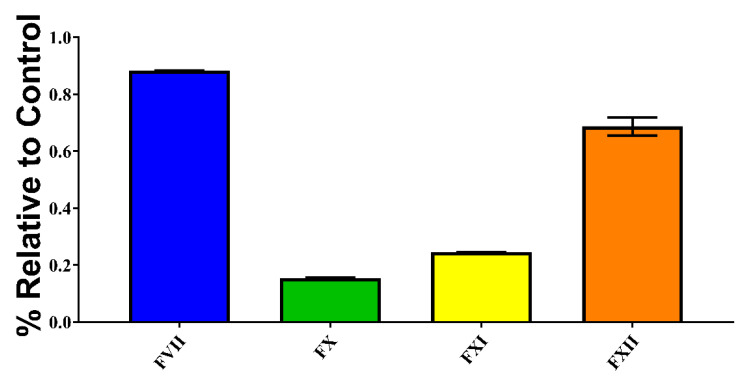
**Factor activation assay showing *P. volcanicum’s* procoagulant ability to activate FVII, FX, FXI, and FXII.** Activation is shown as the relative percentage of zymogen converted to its active form compared with the active-zymogen positive control. Assays were performed in triplicate (*n* = 3) with data representing the mean ± SD.

As *P. volcanicum* was unique in possessing a true procoagulant venom, additional testing was performed to deduce the mechanism responsible for the observed activity. Testing confirmed that *P. volcanicum* did in fact induce clotting in plasma through factor activation. Indeed, four zymogens were activated, with the differential potency of FVII > FXII >> FXI > FX (Figure 3).

**Figure 4:**
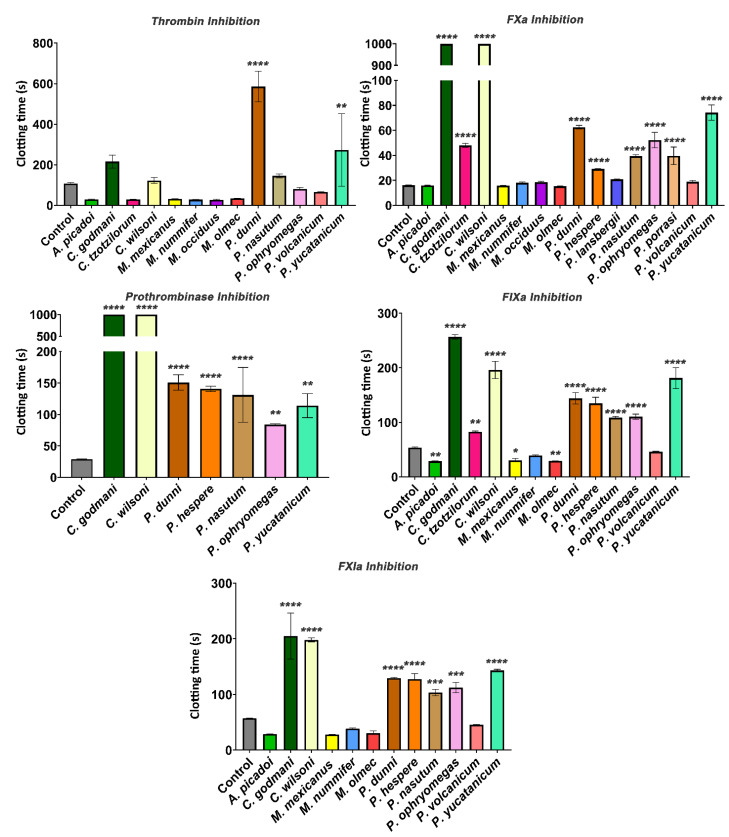
**Coagulation-cascade-factor-inhibition assays showing the inhibitory effects of *Cerrophidion godmani*, *C*. *wilsoni, P. dunni, P. nasutum, P. ophryomegas, and P. yucatanicum* on thrombin, FXa, prothrombinase, FIXa, and FXIa.** Venoms that induced clotting could not be used for the prothrombinase inhibition assay as plasma was clotted before machine recording. Assays with venoms missing were due to a lack of stock, and such venoms were unable to be tested. Assays were performed in triplicate (*n* = 3) with data representing the mean ± SD. Values were statistically analyzed using one-way ANOVAs with a multiple-comparison tests compared with the negative control. Statistical significance from the negative control is indicated by * *p* < 0.1, ** *p* < 0.01, *** *p* < 0.001, or **** *p* < 0.0001.

**Figure 5:**
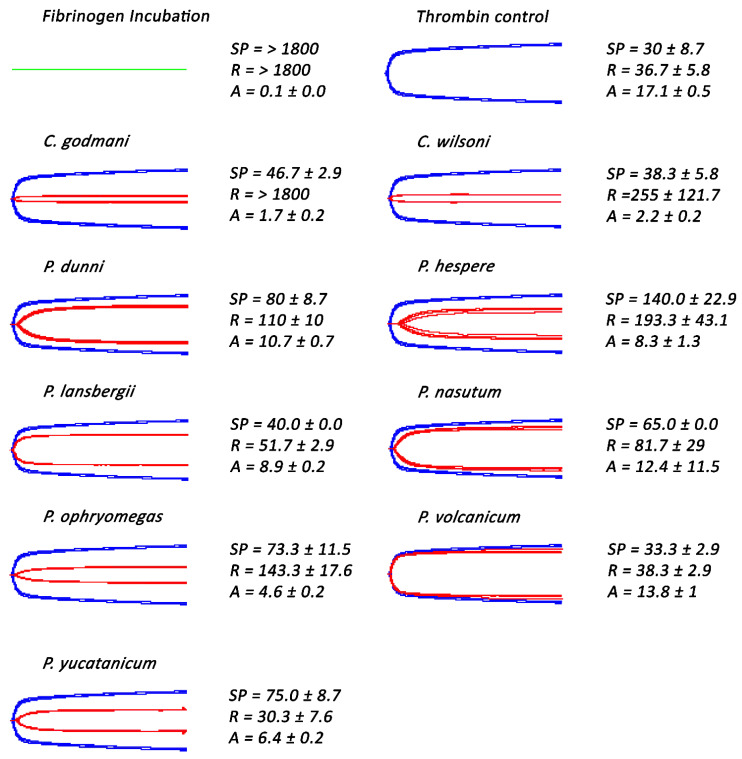
**Thromboelastography traces showing effects of two *Cerrophidion* and seven *Porthidium* species venoms that did not directly clot fibrinogen.** A solution of fibrinogen was incubated for 30 min either alone (for the thrombin control) or with venoms, followed by the addition of thrombin. The green trace represents the 30 min fibrinogen incubation for all samples. Blue traces represent samples in which thrombin was added to fibrinogen in the absence of venom. Red traces represent samples in which venoms were incubated with a fibrinogen solution for 30 min, followed by the addition of thrombin. SP = Split point, i.e., the time taken until a clot began to form (min). R = time to initial clot formation, where formation is 2 mm + (min). A (amplitude) = clot strength (mm). Assays were performed in triplicate (*n* = 3) with data representing the mean ± SD.

Anticoagulant venoms were tested for inhibitory action upon several clotting factors (Figure 4). While *C. godmani* and *C. wilsoni* were the most potent in inhibiting FIXa, FXa, FXIa, and the prothrombinase complex, other species also showed significant inhibition of these clotting enzymes, including, *P. dunni*, *P. nasutum*, *P. ophryomegas*, *P. porrasi,* and *P. yucatanicum* (Figure 4). In contrast, only *P. dunni* showed evidence of strong thrombin inhibition (Figure 4). All anticoagulant venoms tested also showed fibrinogenolytic activity, cleaving the fibrinogen molecule in a destructive (non-clotting) manner and preventing clotting despite the addition of thrombin (Figure 5).

**Figure 6:**
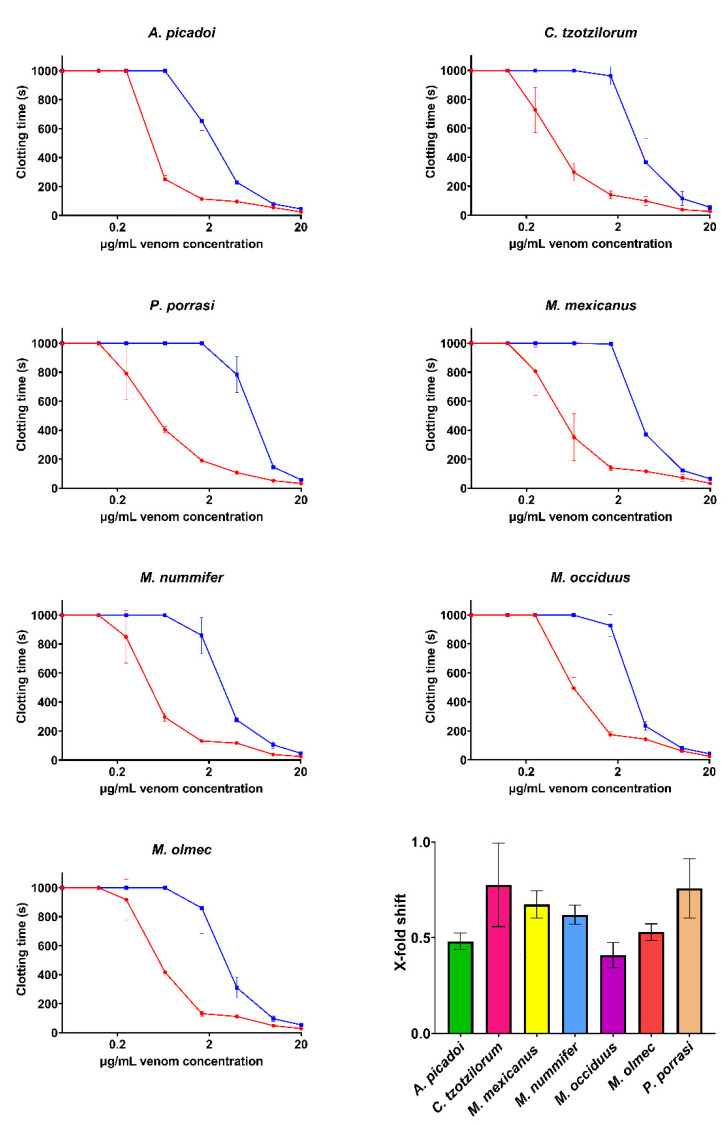
**Concentration–response curves showing the pseudo-procoagulant effects and the relative efficacy of the PoliVal-ICP antivenom.** Curves represent the clotting time of both the venom-only assay (red) and the incubation-with-antivenom assay (blue). The bar graph represents the X-fold shift value of each venom when incubated with antivenom. Calculated values represent antivenom neutralization, where 0 is no neutralization and >0 indicates neutralization. Assays were performed in triplicate (*n* = 3), excluding *P. porrasi*, which was in duplicate (*n* = 2) due to a shortage of venom supply. Data represent the mean ± SD. Note: some data points have error bars that are smaller than the size of the symbol.

**Figure 7:**
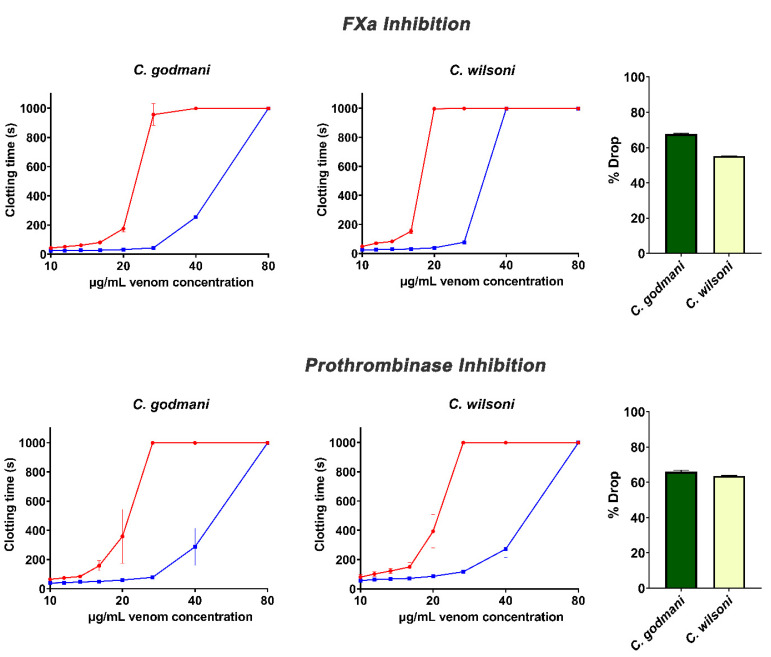
**Concentration–response curves showing the anticoagulant effects of two *Cerrophidion* venoms and the relative efficacy of the PoliVal-ICP antivenom in neutralizing FXa-inhibition and the inhibition of the prothrombinase complex.** Curves represent the venom-only assay (red) and the incubation-with-antivenom assay (blue). Bar graphs indicate the percentage drop-in venom activity of venom incubated with antivenom relative to the assay of venom incubated without antivenom. Assays were performed in triplicate (*n* = 3) with data representing the mean ± SD. Note: some data points have error bars that are smaller than the size of the symbol.

**Figure 8:**
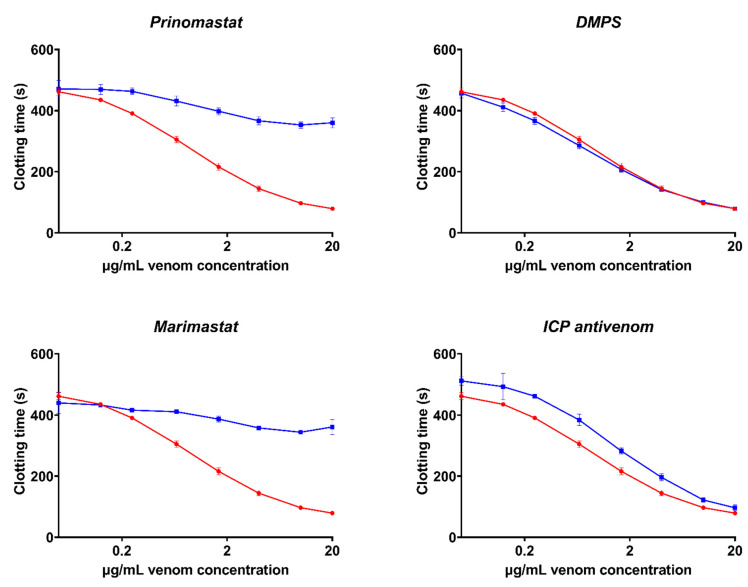
**Concentration–response curves showing the procoagulant effect of *P. volcanicum* venom on plasma and the relative efficacy of three SVMP inhibitors and the PoliVal-ICP antivenom in neutralizing the venom effects.** Curves represent the venom-only assay (red) and the incubation-with-inhibitor/antivenom assay (blue). Assays were performed in triplicate (*n* = 3) with data representing the mean ± SD. Note: some data points have error bars that are smaller than the size of the symbol.

Assays investigating the efficacy of the ICP antivenom demonstrated a significant ability to neutralize the fibrinogen-clotting pseudo-procoagulant activity and the enzyme-inhibiting anticoagulant effects of venom (Figure 6 and Figure 7). However, the ICP antivenom showed poor cross-reactivity towards the procoagulant activity of *P. volcanicum* venom (Figure 8). In contrast, metalloprotease inhibitors prinomastat and marimastat were effective in neutralizing *P. volcanicum*-venom activity, while SVMP inhibitor 2,3-dimercapto-1-propanesulfonic acid (DMPS) performed poorly by comparison (Figure 8).

## 3. Discussion

This study assessed the coagulotoxic effects of sixteen venoms spanning the full ecological, geographical, and taxonomical diversity of the *Atropoides/Cerrophidion/Metlapilcoatlus/Porthidiium* clade, thereby representing the most complete study to date of venom functional activity within this group of snakes. Many of the species included were shown to possess the ability to directly clot fibrinogen, indicating that their venoms act in a pseudo-procoagulant (‘thrombin-like’) manner (Figure 1). This is a common aspect of pit-viper venom that has been described in many species, whereby the venom cleaves fibrinogen to form weak fibrin clots that are easily broken down, resulting in a net anticoagulant effect [25,26,27,28,29]. Further thromboelastographic testing confirmed this pseudo-procoagulant activity, revealing that the venoms that clotted fibrinogen produced weak clots in human plasma (Figure 2).

The results indicate that the most parsimonious explanation is that the pseudo-procoagulant-venom activity is basal to the *Atropoides/Cerrophidion/Metlapilcoatlus* clade, with classic anticoagulant activity arising as a secondary evolution on two separate occasions (*C. godmani* and *C. wilsoni*) (Figure 1). In contrast, classic anticoagulant activity is basal to the *Porthidium* genus, with *P. porrasi* secondarily evolving pseudo-procoagulant-venom activity and *P. volcanicum* secondarily evolving true procoagulant activity. However, the basal condition of the *Atropoides/Cerrophidion/Metlapilcoatlus/Porthidiium* clade as a whole remains uncertain and may be either classically anticoagulant or pseudo-procoagulant. Morphologically the basal trait is a slender/moderately built “*Porthidium*-like” snake, while conversely the extremely robust morphology (epitomized in *Metlapilcoatlus nummifer*) is a derived trait. If venom condition parallels morphology, this suggests that the basal venom condition is “*Porthidium*-like” classic anticoagulant venom (destructive cleavage of fibrinogen and inhibition of activated clotting factors). This indicates that the pseudo-procoagulant (‘thrombin-like’) direct actions on fibrinogen is a derived trait within this clade. Investigating the selection pressures leading to these changes in venom phenotype is a rich area for future research.

The unique procoagulant toxicity of *P. volcanicum* is also a derived state relative to the basal anticoagulant activity. The activity of *P. volcanicum,* whereby it clotted plasma but not fibrinogen, revealed that this venom activated a clotting factor, a trait which is unique within this clade. We report that the activation of both FVII and FXII is largely responsible for the procoagulant activity observed, with FX and FIX activation contributing but to a lesser degree (Figure 3). While Factor X activation is known for *Bothrops* venoms [20], it is only known amongst non-*Bothrops* American pit-viper species from neonate *Crotalus culminatus* as an ontogenetic trait [28]. The FX activation in *C. culminatus* thus represents a convergent evolution relative to *Bothrops* venom. Therefore, FX activation in *P. volcanicum* represents a third independent evolution of this trait within American pit-vipers. Underscoring the usefulness of this trait in prey capture, FX activation has evolved convergently in diverse Afro-Asian snake lineages such as the *Daboia/Macrovipera/Montivipera* viperid clade, viperid species *Bitis worthingtoni*, viperid genus *Cerastes*, viperid genus *Echis*, lamprophiid genus *Atractaspis*, and colubrid genus *Rhabdophis* [30,31,32,33,34,35,36]. In contrast to FX activation being previously widely known as a convergent trait, Factor VII activation by snake venom has only previously been known for the Australian elapid clade of *Oxyuranus* + *Pseudonaja* [37]. As the Australian elapids activate FVII through weaponized form of Factor Xa unique to Australian elapid snake venoms, the ability of *P. volcanicum* to activate FVII thus represents a convergent evolution of this novel trait. In contrast, no snake venom has previously been described as being able to activate Factor XI or Factor XII, thereby underscoring the evolutionary novelty of *P. volcanicum* venom.

The activation of clotting factors is well documented among a wide range of viperid snakes and has been shown to be predominantly driven by the SVMP toxin class [20,28,34]. SVMPs have been shown to represent a large proportion of *P. volcanicum* venom composition [15]. We demonstrated that the clotting ability of *P. volcanicum* was indeed driven by SVMPs through the effective neutralization of venom activity by SVMP inhibitors prinomastat and marimastat. However, the metalloprotease inhibitor DMPS showed poor neutralization of the procoagulant-venom activity (Figure 8). It should be noted that the positive results for prinomastat and marimastat were from idealized (pre-incubation) in vitro conditions and need to be tested in a more dynamic system. Understanding their potential in a clinical setting requires in vivo corroboration. Conversely, the poor response of DMPS under such idealized conditions is not likely to improve in a more dynamic in vivo assay system.

Our findings also demonstrated that the procoagulant-venom effects of *P. volcanicum* were not effectively neutralized by the ICP antivenom. As no similar venom is included within the immunizing mixture, it results in poor cross-neutralization of the complex factor activating the effects of *P. volcanicum* venom. While the ICP antivenom includes a procoagulant phenotype (*Bothrops asper*), it was unsurprising that the antivenom did not cross-react, as *Bothrops* antivenoms are well documented as having limited cross-reactivity even among *Bothrops* species [20,38]. Thus, it would have in fact been surprising if *Bothrops* antivenom cross-reacted with venom from a distantly related (>20 MYA) species that has independently evolved a novel procoagulant-venom phenotype. While the antivenom displayed slight efficacy in isolation against *P. volcanicum*, its use in combination with small-molecule inhibitors may improve the therapeutic potential. Indeed, several recent studies have shown the therapeutic synergy between small-molecule inhibitors and antivenom for a range of snake venoms [39,40].

As previously noted, *Porthidium* species, as well as *C. godmani* and *C. wilsoni*, were potently anticoagulant, and all of them exceeded the machine maximum recording time (Figure 1). Further factor inhibition assays revealed that *C. godmani* and *C. wilsoni* inhibited Factors IXa, Xa, and XIa, and prothrombinase, as did diverse *Porthidium* species (Figure 4). Only *P. dunni* was observed to inhibit thrombin, producing a significant delay in clotting time within the thrombin-inhibition assay (Figure 4). Future work is required to ascertain which toxin types are responsible for inhibiting the clotting factors, with characterized inhibitors of clotting enzymes in viperid-snake venoms such as kunitz peptides, the alpha–beta covalently-linked dimeric-form of lectin toxins, and Group II phospholipase A_2_ toxins [35,41,42,43].

Venom from *C. godmani, C. wilsoni*, and several *Porthidium* species were shown to cleave fibrinogen, contributing to the net anticoagulant effect by depleting the levels of normal, intact fibrinogen usable for fibrin-clot formation (Figure 5). While this is a well-characterized pathophysiological action of venom kallikrein-type serine proteases, the sites of action on fibrinogen are highly variable, with some isoforms being characterized as only cleaving the alpha or beta chain and others as cleaving both [26,27,29,44,45,46,47]. While previous research into the fibrinogenolytic activity of these species has shown *A. picadoi, P. nasutum,* and *P. ophryomegas* venoms to cleave the alpha chain of fibrinogen, leaving the beta and gamma chains unaffected, the alpha-chain cleavage sites have not been determined [48]. For the other species in this study, the specific fibrinogen chains (and sites cleaved within the chain) in which these venoms acted upon remains unknown. Thus, these species warrant further investigation to identify the fibrinogen chain(s) targeted by the venom, including the specific cleavage sites within a particular fibrinogen chain.

Due to the medical significance of bites from these species, the efficacy of the ICP polyvalent regional antivenom was tested on the pseudo-procoagulant (“thrombin-like”) venoms and the anticoagulant *C. godmani* and *C. wilsoni*. The results show the strong neutralization of the pseudo-procoagulant- and enzyme-inhibiting effects of the various venoms, further highlighted by the X-fold shift (Figure 6) and percentage drop (Figure 7). This indicated that the ICP antivenom had a high level of cross-reactivity for species across this diverse group of snakes, despite none of the genera having been used in the immunizing mixture. Our data support the growing body of knowledge surrounding the ICP antivenom’s ability to neutralize venoms from the diverse American pit-vipers [17,21,22]. The efficacy of the ICP polyvalent antivenom on the thrombin inhibiting activity of *P. dunni* was unable to be tested due to venom shortage, and as the pathophysiological target for the remaining *Porthidium* species is unknown, these venoms were not included in the antivenom assays.

In this study, the limited venom supplies restricted antivenom testing to only a single regional product. The ICP antivenom was chosen due to the very wide geographical range of this clade of snakes, which span from Mexico to Central America. The ICP polyvalent product is made with an immunizing mixture containing venoms of *Bothrops, Crotalus,* and *Lachesis* species, while the Mexican bivalent antivenoms are made only using *Bothrops* and *Crotalus*. Thus, the immunologically more complex ICP antivenom was chosen for testing. Future work, however, should investigate the ability of the Mexican bivalent antivenoms to neutralize the snake venoms. Such tests should include the Mexican trivalent antivenom, which, similar to ICP, contains *Bothrops, Crotalus,* and *Lachesis* in the immunizing mixture (and thus is predicted to have a neutralization potential similar to that of ICP) but is a product exported to South America, while the bivalent antivenoms are those primarily used in Mexican hospitals. Due to the more restricted immunological profile, the Mexican bivalent antivenoms may have more limited cross-reactivity, but this must be experimentally determined.

## 4. Conclusions

This study identified a wide variation in coagulotoxic effects in the clade comprising genera *Atropoides, Cerrophidion, Metlapilcoatlus,* and *Porthidium*. The extreme variation in bioactivity was in stark contrast to previous proteomics studies that reported highly similar venoms across the clade. This underscores that proteomics profiles are poor indicators of pathophysiological actions. The most novel finding we reported was the unique factor activation of FVII and FXII as being the major driver of the clotting of plasma by *P. volcanicum* venom. The inhibition of FXa was identified to be largely responsible for the anticoagulant properties of *C. godmani* and *C. wilsoni*, while thrombin inhibition was the case for *P. dunni*. In addition, fibrinogenolytic activity was reported in diverse classically anticoagulant venoms. We observed the effective neutralization of anticoagulant- and pseudo-procoagulant-venom effects when incubated with the ICP antivenom. Metalloprotease inhibitors prinomastat and marimastat were highly effective in neutralizing the procoagulant-venom effects of *P. volcanicum*, while the ICP antivenom and SVMP inhibitor DMPS were not seen to be effective against this activity. Overall, this work supports previous studies, while further adding to the growing body of knowledge surrounding *Atropoides, Cerrophidion, Metlapilcoatlus,* and *Porthidium* venoms and representing the most complete study of their venom functional activity to date. This research study provides a useful platform to uncover specific pathophysiological targets producing venom effects and a means to identify and modernize therapeutic approaches. An important caveat is that the work presented in this study was performed in vitro and should be corroborated by in vivo studies prior to clinical recommendations for use.

## 5. Materials and Methods

### 5.1. Venoms, Plasma, and Reagents

All venom work was conducted under University of Queensland Animal Ethics Approval 2021/AE000075 and UQ Biosafety Committee Approval # IBC/134B/SBS/2015. Human-plasma work was performed under University of Queensland Biosafety Approval #IBC134BSBS2015 and Human Ethics Approval #2016000256. Australian Red Cross (44 Musk Street, Kelvin Grove, QLD 4059, Australia) supplied human platelet-poor plasma (3.2% citrated) under research approval #16- 04QLD-10.

Venom was extracted from sixteen species included within this study: *Atropoides picadoi* (Costa Rica)*, Cerrophidion godmani* (Mexico)*, Cerrophidion tzotzilorum* (San Cristobal, Mexico)*, Cerrophidion wilsoni* (Honduras)*, Metlapilcoatlus mexicanus* (Chiapas, Mexico)*, Metlapilcoatlus nummifer* (Veracruz, Mexico)*, Metlapilcoatlus occiduus* (Mapastepec, Chiapas, Mexico)*, Metlapilcoatlus olmec* (Soteapan, Veracruz, Mexico)*, Porthidium dunni* (Oaxaca, Mexico)*, Porthidium lansbergii* (Colombia)*, Porthidium nasutum* (San Luis de Poitosi, Mexico)*, Porthidium hespere* (Mexico)*, Porthidium ophryomegas* (Costa rica)*, Porthidium porrasi* (Costa Rica)*, Porthidium volcanicum* (Costa Rica)*,* and *Porthidium yucatanicum* (Solidaridad, Quintana Roo, Mexico).

Lyophilized venom was reconstituted in deionized water and then centrifuged (4 °C, 10 min, 14,000 RCF). A working stock of 1 mg/mL and 4 mg/mL for anticoagulant testing was made with 50% double deionized water (DDH_2_O) and 50% glycerol mix to prevent freezing at −20 °C, where it was stored until use to preserve enzymatic activity. Concentrations of the working stock were determined using nanodrop at 280 nm wavelength.

All plasma was stored at −80 °C until use. Reagents used were: Kaolin (Stago catalog # 00597), phospholipid (Stago catalog # 00597), calcium (Stago catalog # 00367), Owren–Koller (OK) buffer (Stago catalog # 00360), factor Xa (Stago catalog # 00311), and thrombin (Stago catalog # 00611).

### 5.2. Coagulation Assays

Coagulation assays were carried out using the Stago STA-R max hemostasis analyzer robot, using assays previously validated by Venom Evolution Lab [31,49]. Pooled human plasma was thawed and warmed to 37 °C in a water bath before being placed into the machine. For testing, venom stocks were manually diluted to 1:10 using Owren–Keller (OK) buffer to obtain 0.1 mg/mL solution before being added to the machine. A series of venom dilutions were automatically carried out by the machine (μg/mL: 0.05, 0.125, 0.25, 0.66, 1.66, 4, 10, and 20). For the 1:1 dilution, 50 µL of the 0.1 µg/mL working stock was then added to 50 µL of calcium, 50 µL of phospholipid, and 25 µL of OK buffer, followed by a 120 s incubation. The robot then added 75 µL of plasma, and the time until clot formation was immediately and automatically measured. If a clot did not formed in 999 s during the assays, the machine automatically stopped, as this was the maximum recording time. The negative control in this study replaced venom with a mix of 50% OK buffer and 50% glycerol; this was used to record healthy plasma spontaneous clotting times.

Venoms that displayed strong coagulotoxic effects were tested against the PoliVal-ICP (Instituto Clodomiro Picado; Costa Rica) antivenom at 50 mg/mL concentration. To identify the target which anticoagulant venoms were acting upon, specific factors were incubated with the venom. Antivenom tests were subsequently performed on assays that showed significant results. The specific-factor-inhibition assays are detailed in Table 1.

To test for SVMP activity on plasma, eight-point dilution curves were run on *P. volcanicum* venom, where proteinase inhibitors DMPS, prinomastat, and marimastat replaced OK buffer as reagents. Prinomastat and marimastat were solubilized in DMSO, followed by dilution to a 10 mM concentration using DDH_2_O. To dilute the concentration to 2 mM, the inhibitor aliquots were thawed and pooled to a 900 μL total volume diluted into 3600 μL of OK buffer. DMPS was solubilized in DMSO and diluted in DDH_2_O to a 20 mM concentration.

### 5.3. Thromboelastography (TEG)

TEG was used to determine the strength and elasticity of the clots formed by the venoms. For anticoagulant venoms, the fibrinogen-destructive properties were also tested. Experiments were performed using methods previously validated by Venom Evolution Lab [26,34,44]. The following assay conditions were run: 7 µL of venom was added to 72 µL of CaCl_2_, 72 µL of phospholipid, 20 µL of Owren-Keller buffer followed by 189 µL of plasma or fibrinogen and then run immediately for 30 min to leave appropriate time for clot formation. For venoms that produced no clots, assays were completed with an additional step by adding 7 µL of thrombin followed by a further 30 min to determine if fibrinogen had been destroyed by the venom to prevent clotting.

### 5.4. Clotting-Factor Activation

Fluoroskan Ascent™ (Thermo Scientific, Vantaa, Finland) was used to determine the activation of clotting factors FVII, FX, FXI, and FXII. Volumes of 10 μL of phospholipid, 10 μL of venom, and 10 μL of zymogen were manually pipetted, followed by the automated pipetting of 70 μL of a mixture of buffer (5 mM CaCl_2_, 150 mM NaCl, and 50 mM Tris-HCl at pH 7.3) and Fluorogenic Peptide Substrate (ES011 substrate Boc-Val-Pro-Arg-AMC; Boc: t-Butyloxycarbonyl; 7-Amino-4-methyl coumarin) into each experimental well of a 384-well plate. Activated factors were used in place of zymogens as positive controls. The zymogen was also replaced in the venom control wells by 10 μL of Fluoroskan buffer to determine the activity of the venom directly upon the substrate. Fluorescence generated by the cleaving of the substrate was automatically recorded by the machine. The results were obtained by subtracting blank values from reactions, followed by the subtraction of venom without zymogen from venom with zymogen.

### 5.5. Statistics

All assays were performed in triplicate, and the data were analyzed using GraphPad prism 9.0 software. Eight-point dilution curves were shown in logarithmic form for ease of viewing. For comparison of the antivenoms ability to neutralize venom activity, the area under the curve (AUC) was calculated for both venom curves and venom + antivenom curves. Subsequently, an X-fold shift and percentage drop was calculated using the following formula:

$$\frac{\mathrm{Venom}+\mathrm{antivenom}\;\mathrm{AUC}}{\mathrm{Venom}\;\mathrm{AUC}}-1$$

where a value of 0 equals no neutralization. A phylogenetic analysis was performed to determine ancestral states. The phylogenetic tree used was based upon Alencar, Quental, Grazziotin, Alfaro, Martins, Venzon, and Zaher [24] and was imported into R using the APE package to analyze the evolutionary patterns across genera.

All data used for statistical material can be found in the Supplementary Material.

## Figures and Tables

**Table 1:** Coagulation-assay protocols.

Fibrinogen-Clotting Assay	Step 1: 50 µL of 0.1 µg/mL Venom (1 mg/mL 50% Glycerol Stock Diluted with OK Buffer) + 50 µL of 0.025 M Calcium + 50 µL of Phospholipid + 25 µL of OK Buffer. Step 2: 120 s Incubation. Step 3: Addition of 75 µL of 4 mg/mL Fibrinogen.
Factor Xa-inhibition assay	Step 1: 50 µL of 0.1 µg/mL venom (1 mg/mL 50% glycerol stock diluted with Owren–Koller (OK) buffer) + 50 µL of 0.025 M calcium + 50 µL of phospholipid + 25 µL of Factor Xa. Step 2: 120 s incubation. Step 3: Addition of 75 µL of plasma.
FIXa inhibition	Step 1: 50 µL of 0.1 µg/mL venom (1 mg/mL 50% glycerol stock diluted with Owren–Koller (OK) buffer) + 50 µL of 0.025 M calcium + 50 µL of phospholipid + 25 µL of Factor IXa (1 mg/mL). Step 2: 120 s incubation. Step 3: Addition of 75 µL of plasma.
FXIa inhibition	Step 1: 50 µL of 0.1 µg/mL venom (1 mg/mL 50% glycerol stock diluted with Owren–Koller (OK) buffer) + 50 µL of 0.025 M calcium + 50 µL of phospholipid + 25 µL of Factor XIa (1 mg/mL). Step 2: 120 s incubation. Step 3: Addition of 75 µL of plasma.
Thrombin-inhibition assay	Step 1: 50 µL of 0.1 µg/mL venom (1 mg/mL 50% glycerol stock diluted with OK buffer) + 50 µL of 0.025 M calcium + 50 µL of phospholipid + 25 µL of thrombin. Step 2: 120 s incubation. Step 3: Addition of 75 µL of 4 mg/mL fibrinogen.
Prothrombinase-complex-inhibition assay	Step 1: 50 µL of 0.1 µg/mL venom (1 mg/mL 50% glycerol stock diluted with OK buffer) + 50 µL of 0.025 M calcium + 50 µL of phospholipid + 75 µL of plasma. Step 2: 120 s incubation. Step 3: Addition of 25 µL of Factor Xa.
Fibrinogen-clotting assay with antivenom	Step 1: 50 µL of 0.1 µg/mL venom (1 mg/mL 50% glycerol stock diluted with OK buffer) + 50 µL of 0.025 M calcium + 50 µL of phospholipid + 25 µL of 5% concentration of ICP polyvalent antivenom diluted with OK buffer. Step 2: 120 s incubation. Step 3: Addition of 75 µL of 4 mg/mL fibrinogen.
Prothrombinase-inhibition assay with antivenom	Step 1: 50 µL of 0.1 µg/mL venom (4 mg/mL 50% glycerol stock diluted with OK buffer) + 50 µL of 0.025 M calcium + 50 µL of phospholipid + 75 µL of plasma + 25 µL of 5% concentration of ICP polyvalent antivenom diluted with OK buffer. Step 2: 120 s incubation. Step 3: Addition of 25 µL of Factor Xa.
FXa-inhibition assay with antivenom	Step 1: 50 µL of 0.1 µg/mL venom (4 mg/mL 50% glycerol stock diluted with Owren–Koller (OK) buffer) + 50 µL of 0.025 M calcium + 50 µL of phospholipid + 25 µL of Factor Xa + 25 µL of 5% concentration of ICP polyvalent antivenom diluted with OK buffer. Step 2: 120 s incubation. Step 3: Addition of 75 µL of plasma.

## Data Availability

All raw data is available in supplementary material.

## Supplementary Materials

The following is available online at https://www.mdpi.com/article/10.3390/toxins14080511/s1, Supplementary File S1, all raw data.
